# Ciprofloxacin Resistance in *Neisseria meningitidis*, France 

**DOI:** 10.3201/eid1408.080040

**Published:** 2008-08

**Authors:** Anna Skoczynska, Jean-Michel Alonso, Muhamed-Kheir Taha

**Affiliations:** * Institut Pasteur, Paris, France; † National Medicines Institute, Warsaw, Poland

**Keywords:** Neisseria meningitidis, ciprofloxacin, resistance, letter

**To the Editor:** Infections with *Neisseria meningitidis* may occur as outbreaks or epidemics. Consequently, chemoprophylaxis for contacts is generally recommended. Ciprofloxacin is frequently used in adults in a convenient 1-dose regimen (*1*). Resistance to this antimicrobial drug in *N*. *meningitidis* is rare (MIC>0.06 mg/L) and has been reported only in sporadic cases in Greece, France, Australia, Spain, Argentina and Hong Kong Special Administrative Region, People’s Republic of China (*2*–*5*). However, recent reports have described ciprofloxacin-resistant (Cip-R) serogroup A meningococci from 2 outbreaks in Delhi, India (*6*), and a cluster of 3 serogroup B meningococci in the United States (*7*). This information is of concern because of the high epidemic potential of serogroup A isolates, lack of vaccine against serogroup B meningococci, and possible horizontal Cip-R gene transfer to other meningococcal isolates.

Experimental work was conducted in the *Neisseria* Unit of the Institut Pasteur in Paris. We screened all clinical *N*. *meningitidis* isolates received at the French National Reference Center for Meningococci in Paris since 1999 for ciprofloxacin resistance. Of these isolates, 4,900 were from France and 246 were from African countries (Burkina Faso, Cameroon, Central African Republic, Côte d’Ivoire, Madagascar, Niger, Rwanda, Senegal, and Tunisia). Only 3 isolates tested were resistant to ciprofloxacin (MICs = 0.19 mg/L), and all were isolated from cases of invasive disease in France.

Two serogroup A, serotype 4, serosubtype P1.9, Cip-R isolates belonged to different sequence types (STs), ST-7 (Cip-R1) and ST-4789 (Cip-R2), although they belonged to the same clonal complex (ST-5/subgroup III). Cip-R1, which showed decreased susceptibility to penicillin, was isolated in 2004 from the blood of a 7-year-old girl. This isolate was most likely imported from Africa. Cip-R2 was isolated from the cerebrospinal fluid of a 77-year-old man who had arrived in France from India in 2006. The ST of this isolate (ST-4789) is the same as the ST of isolates from an outbreak in Bangladesh and similar to isolates from an outbreak in India (*6*; http://neisseria.org/nm/typing/mlstdb). Cip-R3 (serogroup W-135, nontypeable, subtype P1.5), which was isolated from blood and cerebrospinal fluid of an 82-year-old woman in 2006, belonged to a new ST (ST-6361). Current ciprofloxacin resistance has not been documented among invasive W-135 meningococcal isolates since 2 W-135 resistant meningococci were isolated from sputum samples of elderly patients in Spain (*3*).

To investigate the mechanism of resistance in the isolates, fragments of *gyrA* (847 bp) and *parC* (822 bp) genes were amplified by using primers gyrA-1F (5′-gttttcccagtcacgacgttgtaATGACCGACGCAACCATCCGCCAC-3′) and gyrA-1R (5′-ttgtgagcggataacaatttcCCAGCTTGGCTTTGTTGACCTGATAG-3′), and parC-1F (5′-gttttcccagtcacgacgttgtaATGAATACGCAAGCGCACGCCCCA-3′) and parC-1R (5′-ttgtgagcggataacaatttcGGAATTGGCGTTCGGCGGCAGCTC-3′), respectively (sequences in lower case letters are adaptors for universal forward and reverse sequences were added for sequencing after amplification). Primers used for amplification of the *parE* gene were as described (*8*).

Sequencing of fragments of *gyrA*, *parC*, and *parE* genes showed a mutation in the *gyrA* gene in the 3 Cip-R isolates resulting in a Thr91 → Ile substitution. Cip-R1 also showed additional alterations of Asn103 → Asp, Ile111 → Val, and Val120 → Ile, which were described for meningococcal isolates (*3*). Sequences of *parC* and *parE* genes were the same as in a ciprofloxacin-susceptible isolate tested. The association of the Cip-R phenotype with mutations in *gyrA* was confirmed by transformation into the susceptible isolate by using appropriate PCR products (*9*). In addition to the common Thr91 → Ile substitution, the 3 Cip-R isolates were distinguishable by additional *gyrA* alterations or phenotypic and genotypic characteristics. This finding suggests independent events and argues against clonal expansion of Cip-R meningococci.

Serogroup A meningococcal isolates in France are rare and mostly imported. Lack of detection of ciprofloxacin resistance among African isolates tested in this study may be caused by the relatively low number of these isolates (n = 246). Therefore, surveillance of antimicrobial drug susceptibility of meningococcal isolates should be enhanced by using molecular approaches that can also be used as nonculture techniques. This molecular approach will be useful in countries with limited access to classic microbiologic culture–based methods. Reports of invasive cases caused by W-135 Cip-R meningococci should alert physicians who use quinolones to treat respiratory infections in elderly persons. This age group is affected most often by invasive meningococcal pneumonia and 54.5% of such cases are caused by W-135 meningococci (*10*).
